# Testing the Steady-State Fluctuation Relation in the Solar Photospheric Convection

**DOI:** 10.3390/e22070716

**Published:** 2020-06-28

**Authors:** Giorgio Viavattene, Giuseppe Consolini, Luca Giovannelli, Francesco Berrilli, Dario Del Moro, Fabio Giannattasio, Valentina Penza, Daniele Calchetti

**Affiliations:** 1 Università degli Studi di Roma “Tor Vergata”, Via della Ricerca Scientifica 1, 00133 Rome, Italy; giorgio.viavattene92@gmail.com (G.V.); luca.giovannelli@roma2.infn.it (L.G.); francesco.berrilli@roma2.infn.it (F.B.); dario.delmoro@roma2.infn.it (D.D.M.); valentina.penza@roma2.infn.it (V.P.); daniele.calchetti@roma2.infn.it (D.C.); 2 INAF—Istituto di Astrofisica e Planetologia Spaziali, Via del Fosso del Cavaliere 100, 00133 Rome, Italy; 3 Istituto Nazionale di Geofisica e Vulcanologia, Via di Vigna Murata 605, 00143 Rome, Italy; fabio.giannattasio@ingv.it

**Keywords:** fluctuation theorems, non-equilibrium stationary state, convection, astrophysical fluid dynamics, Sun, photosphere, spectroscopy

## Abstract

The turbulent thermal convection on the Sun is an example of an irreversible non-equilibrium phenomenon in a quasi-steady state characterized by a continuous entropy production rate. Here, the statistical features of a proxy of the local entropy production rate, in solar quiet regions at different timescales, are investigated and compared with the symmetry conjecture of the steady-state fluctuation theorem by Gallavotti and Cohen. Our results show that solar turbulent convection satisfies the symmetries predicted by the fluctuation relation of the Gallavotti and Cohen theorem at a local level.

## 1. Introduction

Non-equilibrium dynamical systems are quite ubiquitous in nature, and are due to non-conservative forces acting on open systems, which imply a continuous dissipation generally transferred to other systems (*thermostats*) in the form of heat. If the feeding of energy by the non-conservative forces to these systems is continuous, then after a transient phase these systems get a *stationary condition*; i.e., they get a *non-equilibrium stationary state* (NESS), which is characterized by a time-invariant distribution in the system phase-space. The dynamics of such systems are generally characterized by the occurrence of large fluctuations, which are related to the non-conservation (contraction) of the system phase-space. In particular, dynamical systems near a NESS are characterized by a non-vanishing entropy production rate, which manifests the tendency of the system to relax towards the equilibrium [1,2]. The entropy production rate σ(t) is, indeed, the physical parameter that describes the spontaneous tendency of a non-equilibrium physical systems to evolve towards equilibrium.

Several different approaches have been proposed to characterize the emergence of such large fluctuations of time-averaged entropy production rates in non-equilibrium systems. Among those, the fluctuation theorem by Gallavotti and Cohen (GC) [3,4] leads to interesting predictions. Indeed, the GC fluctuation theorem states that the probability distribution of the phase space contraction $p\sigma_{+}$ over large time span τ satisfies a non-trivial symmetry property [5]; i.e.,

(1) $$\lim_{\tau \to \infty }\frac{1}{\tau \sigma_{+}}\ln\frac{\pi_{\tau }\left(+p\right)}{\pi_{\tau }\left(-p\right)}=p,$$

where $\sigma_{+}$ is the average phase space contraction rate over an infinite time, and *p* is a dimensionless characterization of the phase space contraction (${\langle p\rangle }_{\tau \to \infty }=1$). Although fluctuation relations are intrinsically related to systems characterized by reversible dynamics, it has been shown that under specific hypotheses, Equation (1) remains also valid for systems near a nonequilibrium stationary state showing an irreversible evolution at a macroscopic scale (for an extended discussion, see, e.g., [2,6,7,8,9]). The validity of the fluctuation relation for evolutions modeled at a macroscopic level by irreversible equations is based on possible properties of the evolution on the attracting set in the phase space (the validity of the chaotic hypothesis, the identification of phase space contraction with entropy production rate and a symmetry of time reversal for motions restricted to the attracting set that in some cases can follow from the microscopic time reversal symmetry), as clearly discussed in a recent work by Gallavotti [8,9]. Furthermore, because the quantity $p\sigma_{+}$ in Equation (1) can be interpreted as an average entropy production rate, the fluctuation relation in Equation (1) quantifies the breaking of the entropy production rate symmetry at large τ, providing a relationship between the probability of observing positive and negative entropy fluctuations in NESS. More in general, because the fluctuation relation of GC theorem (GCFR) deals with the statistical features of a variable related to the phase space contraction rate, it has been assumed to be valid in the case of flux quantities, *J* (e.g., heat, energy or momentum flux) [10]. In this case the FR can be equivalently written, as follows,

(2) $$\lim_{\tau \to \infty }\frac{1}{\tau }\ln\frac{\pi (J_{\tau }=J)}{\pi (-J_{\tau }=-J)}=\alpha_{+}J,$$

where $\alpha_{+}$ is a constant with dimension inverse of time times $J^{-1}$.

Furthermore, although the GCFR was originally derived for global variables, it was later empirically extended also in time averaged quantities, such as [11,12]:

(3) $$J_{\tau }\left(r\right)=\frac{1}{\tau }\int_{t}^{t+\tau }J\left(r,t^{\prime }\right)dt^{\prime },$$

where r is the spatial position. From Equation (2), the quantity α(τ) acquires a linear dependence on τ,

(4) $$\ln\frac{\pi (+J_{\tau })}{\pi (-J_{\tau })}∼\alpha_{+}\tau J_{\tau },$$

which is expected to be valid in an asymptotic regime (τ→∞), and thus,

(5) $$\alpha \left(\tau \right)∼\alpha_{+}\tau$$

for a system that satisfies the symmetries predicted by the GC Fluctuation Theorem at a local level.

Much work has been done on both the numerical and the experimental side to test the validity of GC fluctuation theorem [10,13,14]. For instance, Ciliberto et al. [10] proved the validity of the GCFR with a von Karman experimental setup (two counter rotating plane-parallel disks) and in a wind tunnel experiment. Furthermore, Shang et al. [13] verified the validity of the GCFR in a controlled convection experiment at high Rayleigh number (up to ∼$10^{9}$). Although solar convection is characterized by much higher Rayleigh numbers, at least three orders of magnitude higher, this latter case establishes a reference for our analysis.

In the framework of astrophysical systems, the solar intensity pattern (e.g., granulation) is a manifestation of a non-equilibrium phenomenon, the solar convection, which occurs in the outer layers of the Sun. The solar turbulent convection covers a wide range of spatial and temporal scales, and it is usually described in terms of different phenomena; i.e., granulation, mesogranulation and supergranulation. Nonetheless, it exhibits properties that continuously extend over the different scales, suggesting that solar convection requires a comprehensive analysis for its understanding (see, e.g., [15,16,17,18,19,20,21,22,23,24,25,26]). The granular scale is the spatial scale where most of the energy carried by the convection is delivered in the photosphere. Detailed spectropolarimetric observations of the photospheric lines along the solar surface and its time evolution tightly constrain the properties of the solar convection in terms of spatial and temporal scales, plasma upflows and downflows, as well as its horizontal motion and its thermodynamical properties. Granular convective cells have typical diameters of ∼1 Mm, bright and thus hot central areas, where upflows are located (∼1 km s^−1^), and dark, and thus cooler, lanes, that separate adjacent convective cells and where downflows (∼−1.5 km s^−1^) are located (e.g., [27,28,29,30,31,32,33,34] and references therein). The emergence of distinct scales of plasma motion on larger scales—mesogranulation [35] and supergranulation [36,37,38]—is highlighted by spatio-temporal filters applied by the observation techniques (e.g., time averaging) and by tracking the horizontal motion of the plasma. However, the radial component of the solar convection dynamics is dominated by the granular scale.

Turbulent convection, responsible for the solar intensity pattern, is driven largely by radiative cooling from the photosphere, the solar layer from which most photons can escape to space. These radiative losses, and therefore entropy losses, in the photosphere are an “entropy well” for the entropy produced in the solar nucleus, which is the “entropy source” inside the Sun. The removed entropy from the photospheric plasma creates overdense, turbulent fluid plumes which penetrate into the Sun under the action of gravity. These overdense, low entropy, plumes basically drive solar turbulent convection, leaving the underdense, high entropy, hot plasma parcels in a secondary role [39,40]. In other words, buoyancy acts on both cold (low entropy) and hot (high entropy) plasma parcels, but the convection process is dominated by low entropy plasma that penetrates the interior of the Sun. It is worth noting that we can neglect total entropy change in the Sun for the considered time interval, because our star is in a nonequilibrium steady state.

Furthermore, convection on the Sun is the only case in which we can observe the 3D properties of this process over time in a star, and that is crucial for the development of stellar convection theories (see [41,42]).

In this work, we report on a study of the statistics of the fluctuations of the local heat flux in the solar quiet photosphere (i.e., the regions where the effect of the magnetic field is negligible), which is a natural laboratory for investigating the physics of turbulent plasma convection at very high Rayleigh numbers (≥$10^{12}$) [25,40,43,44,45]. The spectro-polarimetric high resolution images, acquired by ground-based telescopes, allow us to inquire the solar convection at photospheric level with unprecedented detail. In fact, by using these observations, we can evaluate the vertical heat flux from the solar surface temperature and the vertical velocity and use it as a proxy of the entropy production rate, or more generally, the phase space contraction rate [5,13].

## 2. Dataset and Methods

**Figure 1:**
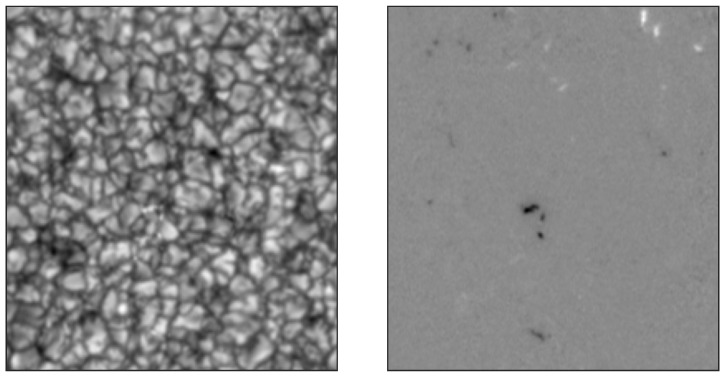
**Left panel**: sample map of the Stokes I intensity of the IBIS/DST dataset in the continuum near the Fe I 630.15 nm spectral lines. **Right panel**: sample map of the Stokes V left lobe of the Fe I 630.15 nm spectral line. The field of view is ∼25 × 25 Mm^2^.

The dataset used to perform this analysis was acquired at the National Solar Observatory (NSO, Sacramento Peak, New Mexico) on 21 November 2006 using the Dunn Solar Telescope (DST) and the Interferometric BIdimensional Spectropolarimeter (IBIS [46,47]). The IBIS instrument acquires bidimensional monochromatic images by performing imaging spectral scans with a spectral passband of ≃4 pm. The field-of-view (FoV) imaged by IBIS is approximately 25 × 25 Mm^2^ on the solar photosphere (corresponding approximately to 40 × 40 arcsec^2^ of angular FoV). In spectro-polarimetric mode, IBIS can also measure the four Stokes profiles of the incoming light (the total intensity *I*, the excess of vertical/horizontal linear polarization *Q*, the excess of 45 degrees linear polarization *U* and the excess of the circular polarization *V*) of the selected spectral lines. During this observational campaign, IBIS acquired the Stokes profiles and the broadband images in the spectral region containing the Fe I spectral line at 630.15 nm. The DST was pointed at the solar disk center, so in this dataset the vertical (radial) direction coincides with the line-of-sight (LoS) direction. Accordingly, all quantities computed as LoS components are indicated as z components. The pixel scale is ≃130 km on the solar photosphere (corresponding to an angular resolution of 0.18 arcsec), the temporal resolution is 89 seconds (the time needed to perform a complete spectral scan) and the dataset consists of 41 spectral scans in total; therefore, the whole duration of the dataset is approximately one hour. A sample map of the Stokes I intensity is reported in Figure 1 (left panel): the typical photospheric convection pattern with the solar granulation is clearly visible. A sample map of the Stokes V, i.e., the excess of circular polarization, is reported in Figure 1 (right panel). Stokes V maps can be used to compute the z component of the magnetic field ($B_{z}$) present in the solar photosphere. Qualitatively, black and white regions corresponds to positive and negative $B_{z}$, respectively. More details and information about the dataset can be found in [48,49,50,51]. The dataset has been calibrated using the standard IBIS pipeline, as described in [52]. Following Shang et al. [13] and assuming that the heat transport occurs mainly in the vertical direction, the entropy production rate σ(r,t) can be directly associated with the vertical heat flux $j_{z}\left(r,t\right)$:

(6) $$\sigma \left(r,t\right)\propto V_{0}\;j_{z}\left(r,t\right)\;{▽}_{z}\left(\frac{1}{T}\right),$$

where $V_{0}$ is the volume in which we evaluate the local properties, ${▽}_{z}$ is the vertical gradient and *T* is the temperature. We assume that $V_{0}$ and ${▽}_{z}\left(\frac{1}{T}\right)$ are constant and equal for all the pixels in the FoV.

**Figure 2:**
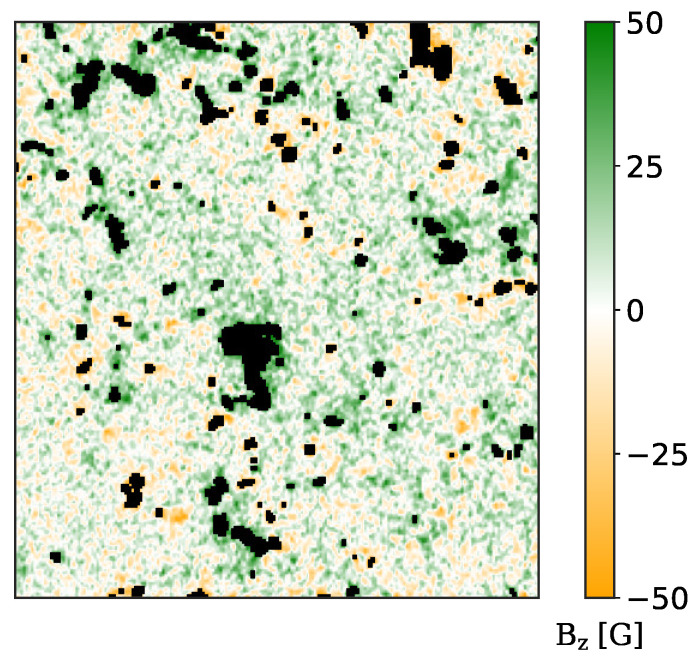
Sample map of the magnetic field intensity (vertical component) evaluated using the CoG method applied to the Fe I 630.15 nm spectral line. Black pixels have a magnetic field intensity greater than 50 G and are excluded from our analysis.

Solar convection is inhibited by strong magnetic fields (e.g., in sunspots). We are interested in the statistical properties of the solar convection in a regime where it dominates over magnetic effects; i.e., the so called Quiet Sun. In the case of granulation, equipartitioning of magnetic energy density and kinetic energy density is reached at ∼500 G. Nonetheless, simulations [53] showed that convection smoothly transits trough different regimes as the magnetic field intensifies: from convection-dominated to magnetoconvection, arriving to the convection inhibition. Transition to magnetoconvection seems to happen at a value of 50 G. Thus, we mask out and we exclude from our analysis the areas with a magnetic flux intensity signal greater than 50 G, computed using the center of gravity (CoG) [54] method on the Stokes V profiles of the 630.15 nm spectral line. Furthermore, a threshold of 50 G excludes also pixels with unresolved strong magnetic fields phenomena. Left and right-handed circular polarized light ($I_{\pm }$), in the proximity of the 630.15 nm line, exhibits lobes, whose spectral distance is proportional to the z component of the magnetic field. The CoG method is based on the measurements of the displacements of the centroids of the two lobes. The z component of the magnetic field $B_{z}$, expressed in G, is evaluated using the following equation:

(7) $$B_{z}=\frac{1.071\times 10^{9}}{\bar{g}\lambda_{0}^{2}}\left(\lambda_{+}-\lambda_{-}\right)$$

where $\lambda_{0}$ is the center of the observed wavelength in the rest frame and is expressed in Angstrom; $\bar{g}$ is the Landé factor; and $\lambda_{+}$ and $\lambda_{-}$ are respectively the spectral positions of the positive and the negative lobes of the Stokes V profile:

(8) $$\lambda_{\pm }=\frac{\int \left[I_{c}-I_{\pm }\left(\lambda \right)\right]\lambda \;d\lambda }{\int \left[I_{c}-I_{\pm }\left(\lambda \right)\right]d\lambda }$$

where $I_{c}$ is the intensity of the unpolarized continuum, λ is the wavelength and the integrals are extended over the entire line profile. A sample of the magnetic field maps is reported in Figure 2.

**Figure 3:**
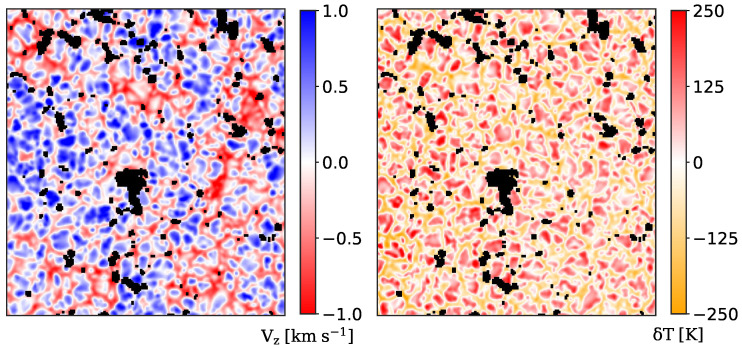
**Left panel**: sample map of the vertical velocity evaluated using the CoG method applied to the Fe I 630.15 nm spectral line. **Right panel**: sample map of the temperature fluctuations evaluated using the Stefan–Boltzmann black-body radiation law applied to broadband images. Black pixels have a magnetic field intensity greater than 50 G and are excluded from our analysis.

The local vertical heat flux is evaluated from vertical velocity maps and temperature maps, as discussed in [55,56]. We compute the vertical velocity maps $v_{z}\left(r,t\right)$ using the CoG method:

(9) $$v_{z}\left(r,t\right)=\frac{\int I(r,t)\lambda d\lambda }{\int I(r,t)d\lambda }$$

where the integral is extended over the entire line profile. A sample of the velocity maps is reported in Figure 3 (Left panel). The temperature fluctuations δT(r,t), with respect to the mean photospheric temperature, are computed from the broadband images by applying the Stefan–Boltzmann black body radiation law [57,58]:

(10) $$\delta T\left(r,t\right)=T_{ref}\left(\sqrt[{4}]{\frac{I(r,t)}{\bar{I}\left(t\right)}}-1\right)$$

where $T_{ref}=5780$ K is the average temperature of the solar photosphere (assuming the Sun as a black body) and $\bar{I}\left(t\right)$ is the average intensity for each spectral scan. A sample of the temperature fluctuation maps is reported in Figure 3 (right panel). We assume that the evaluated temperature fluctuations are associated with the base of the solar photosphere within the photon mean free path (≈70 km [59]), considering that we make use of broadband images to compute them via the Stefan–Boltzmann law.

**Figure 4:**
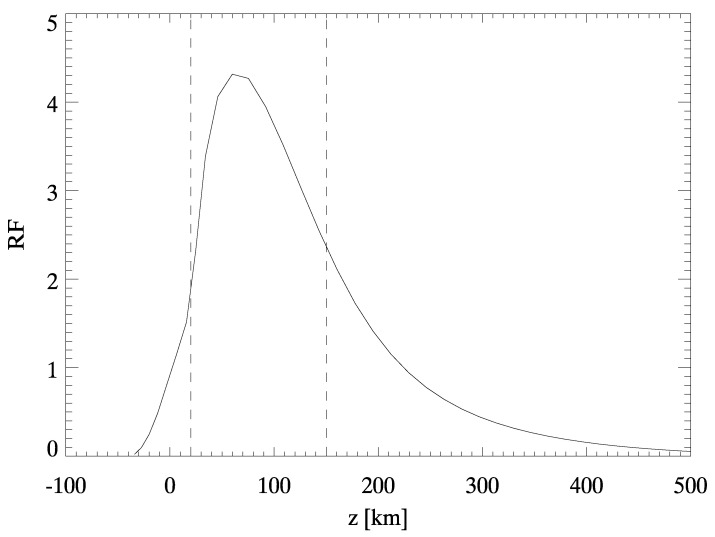
Response function (RF) of the vertical velocity for the Fe I 630.15 nm spectral line, averaged over the sampled spectral points of the observation. Adapted from [55].

As discussed, we use line intensity profiles to derive with the CoG method the vertical velocity maps. We underline here that line profiles are an integral measure of the radiation sources and sinks along the line of sight, and carry the information of many layers of the solar atmosphere. In the case of the Fe I spectral line at 630.15 nm the information mostly comes from the photospheric layers, and we can quantify where the line is more sensitive to velocity variations using the response functions (RFs) [60,61,62,63,64]. Considering small perturbations of the atmospheric parameters (e.g., vertical velocity, magnetic fields, density and so on), the RFs identify the atmospheric layer where the Stokes profiles are more sensible to these perturbations; therefore, the RFs localize the atmospheric layers where the spectral line is mainly formed [64]. Using the RF for the velocity computed for the 630.15 nm spectral line (see Figure 4), we estimate that the atmospheric layer associated with velocity maps is $70_{-50}^{+80}$ km above the base of the photosphere [55]. Thus, due to the temperature fluctuations associated with the base of the photosphere (i.e., ±70 km computed from a Kurucz model [65]), we can assert that the temperature and vertical velocity signals are generated by the same atmospheric layer within the uncertainties.

The velocity and temperature maps are treated with a subsonic $k_{h}-\omega$ filter in order to remove the acoustic oscillations signal of the solar photosphere [66]; this technique is commonly employed to separate the fluctuations due to solar acoustic waves with respect to the fluctuations generated by the convection.

We follow the methodology described in [13] to compute the local vertical heat flux $j_{z}\left(r,t\right)$ (namely, the vertical heat-flux per unit surface). We use the velocity maps $v_{z}\left(r,t\right)$ and the temperature fluctuation maps δT(r,t), evaluated at the same atmospheric layer and within the same pixel, i.e., the same **r**) to compute:

(11) $$j_{z}\left(r,t\right)=\rho \;C_{V}\;v_{z}\left(r,t\right)\;\delta T\left(r,t\right),$$

where ρ is the solar photospheric density and $C_{V}$ the specific heat capacity at constant volume. Equation (11) is a modified version of a phenomenological law, where the thermodynamic force is assumed to be δT(r,t) instead of the gradient of the corresponding intensive quantity (i.e., ▽T). The equivalence δT∝▽T can be justified by the assumption that positive thermal fluctuations, δT>0, associated with upward velocities ($v_{z}>0$), are representative of positive (upward) heat flux (see also, for a more extended discussion [67,68]). Furthermore, in Equation (11) we assume ρ and $C_{V}$ as a constant quantities, as the range of variability of those quantities lies on longer temporal scales respect to the observation duration. For further discussion, see [55]. Therefore, the heat flux in Equation (11) can be written as:

(12) $$j_{z}\left(r,t\right)=\rho \;C_{V}\;j_{z}^{\prime }\left(r,t\right),$$

where $j_{z}^{\prime }\left(r,t\right)=v_{z}\left(r,t\right)\;\delta T\left(r,t\right)$. Thus, the study of heat flux fluctuations can be limited to study the fluctuations of $j_{z}^{\prime }\left(r,t\right)$. To study the statistics of the $j_{z}^{\prime }$ and to test the steady state of the GCFR we computed a running average of $j_{z}^{\prime }$ over a time interval τ:

(13) $$J_{\tau }\left(r,t\right)=\frac{1}{\tau }\int_{t}^{t+\tau }j_{z}^{\prime }\left(r,t^{\prime }\right)dt^{\prime }.$$

## 3. Data Analysis and Discussion

**Figure 5:**
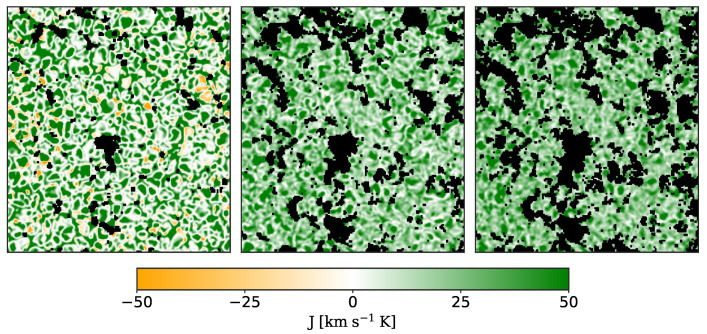
**Left panel**: sample of vertical heat flux maps $J_{1}\left(r,t\right)$ (89 s average) evaluated using Equation (11). Central panel: sample of $J_{12}\left(r,t\right)$ maps (1068 s average). **Right panel**: sample of $J_{24}\left(r,t\right)$ maps (2136 s average). Black pixels have a magnetic field intensity greater than 50 G and are excluded from our analysis. See text for details on the J maps definition.

We evaluate the vertical heat flux pixel by pixel using Equation (11). This local measure leads to a large statistics, with more than $3\times 10^{4}$ samples for each scan. An example of the vertical heat flux maps, for one of the temporal steps, is reported in Figure 5 (left panel).

**Figure 6:**
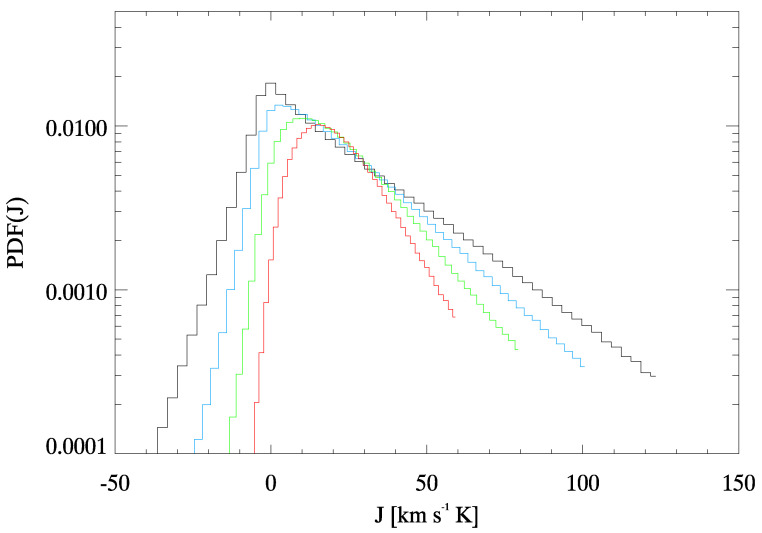
PDFs of the $J_{\tau }$ evaluated with the Kernel method: black is for $J_{2}$ (178 s average), light blue for $J_{4}$ (356 s average), green for $J_{8}$ (712 s average) and red for $J_{16}$ (1424 s average).

Using Equation (13) we compute the various vertical heat fluxes $J_{\tau }\left(r,t\right)$ over the different time steps interval τ: then, $J_{1}\left(r,t\right)$ is the vertical heat flux for each single spectral scan, $J_{2}\left(r,t\right)$ is the one averaged between two spectral scans, $J_{3}\left(r,t\right)$ between three spectral scans and so on. In Figure 5, we report a map of $J_{12}\left(r,t\right)$ (central panel) and a map of $J_{24}\left(r,t\right)$ (right panel). As expected, the convection pattern is smoothed as τ increases. In addition, there are more black (excluded) pixels when we increase the time span of the average due to the evolution and the motion of the magnetic features. The probability density functions (PDFs) of $J_{\tau }$ for each fixed τ are evaluated using the Kernel method [69] and some of them are reported in Figure 6.

The PDFs of the $J_{\tau }$ at each τ show a clear departure from the Gaussian shape, being indeed non-Gaussian, asymmetric and kurtotic. This confirms that we do not deal with an equilibrium and/or near equilibrium fluctuation process, where the PDFs are expected to follow a quasi-Gaussian distribution. Conversely, the observed functional form of the vertical heat-flux proxy resembles the lepto-kurtotic distributions observed in several turbulent phenomena [10]. Furthermore, the PDFs shrink going from $J_{1}$ to $J_{29}$, and so τ increases, while the peak of the PDF tends to a non-zero value for τ→∞, as it should be for a non-equilibrium process with a positive entropy production rate.

The limiting non-zero value of the local vertical heat flux is $j_{z}∼200$ kW m^−2^, assuming that the solar photospheric plasma density is $\rho ∼3\times 10^{-4}$ kg m^−3^ (from the solar Kurucz model [65]) and the specific heat capacity is $C_{V}∼10^{4}$ J kg^−1^ K^−1^.

**Figure 7:**
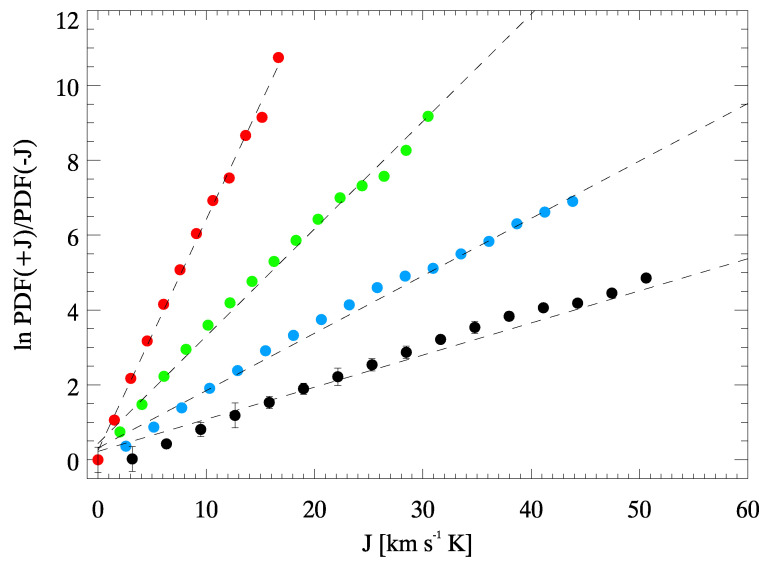
Dependence of ln PDF$\left(\pm J_{\tau }\right)$ on $J_{\tau }$ evaluated using Equation (2). We follow the same colors of Figure 6: black is for $J_{2}$ (178 s average), light blue for $J_{4}$ (356 s average), green for $J_{8}$ (712 s average) and red for $J_{16}$ (1424 s average). The dashed lines are the relative linear fits. Error bars are computed from the number of the samples in each bin in Figure 6, and given the large statistic, are smaller than the circle marks in most of the cases.

In Figure 7 we show the logarithmic ratios of the PDF$\left(\pm J_{\tau }\right)$ as a function of *J* for the same PDFs shown in Figure 6. We also plot the relative linear fits as dashed lines. We can notice that a clear linear dependence is recovered as predicted from GCFR (see Equations (4) and (5)) and that the slope α(τ) steepens as τ increase.

**Figure 8:**
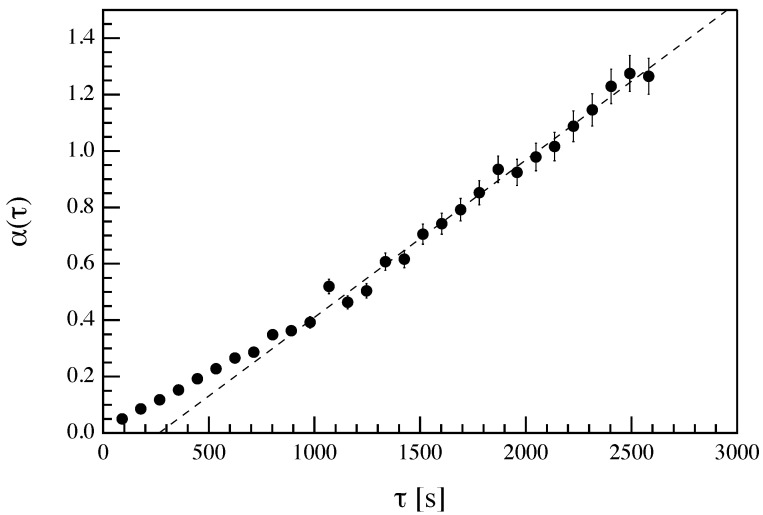
Behavior of α(τ) on timescale τ. The dashed line is a linear best fit in the range τ>900 s.

In Figure 8 we report α(τ) obtained by fitting the logarithmic ratios of the PDF$\left(\pm |J_{\tau }|\right)$ assuming a linear dependence on $J_{\tau }$. Following the previous considerations and if the Equation (4) holds, the α(τ) should be linearly dependent on τ (see also [10,13]); i.e.,

(14) $$\alpha \left(\tau \right)=\alpha_{+}\tau +\beta .$$

This expression is, indeed, not in contrast with Equation (4) because that equation is expected to be valid in the asymptotic limit. Figure 8 clearly shows that for timescales longer than $\tau_{0}∼600÷800$ s a linear dependence of α(τ) on τ is found. Since we are interested in the asymptotic behavior, we perform a linear fit of α for large values of τ (>900 s) and we obtained: $\alpha_{+}=\left(560\pm 10\right)\times 10^{-6}$ km^−1^ K^−1^ and $\beta =\left(-15\pm 1\right)\times 10^{-2}$ km^−1^ s K^−1^.

By re-scaling the $\alpha_{+}$ using the asymptotic value of *J*, i.e., $J_{\infty }∼20$ km s^−1^ K, we can get information on the typical dissipation scale $\tau_{diss}∼{\left(\alpha_{+}^{\prime }\right)}^{-1}$, where $\alpha_{+}^{\prime }=J_{\infty }\;\alpha_{+}$ [8]. In this case we obtain a dissipation scale $\tau_{diss}∼100-120$ s. This timescale is in the same range of timescales associated with the average lifetime of excess thermal energy release and velocity field decorrelation time near the solar surface [28,70]. Indeed, the thermal adjustment time $\tau_{adj}$, i.e., time necessary to release the excess thermal energy, is of a few minutes, and the correlation time of the vertical velocity field at the solar photosphere is of the order of 130 s. [28].

## 4. Conclusions

In this work, we have investigated the validity of the symmetries predicted by the Gallavotti–Cohen fluctuation relation for non-equilibrium systems in the case of the solar turbulent convection by studying the statistics of the local vertical heat flux at different timescales. The local vertical heat flux has been evaluated from vertical velocity maps and temperature maps, which have been computed with the CoG method and the Stefan–Boltzmann law applied to IBIS high-resolution spectro-polarimetric data, respectively. We show that the PDFs of the local vertical heat flux are clearly non-Gaussian, asymmetric and have a non-zero mean value, which confirms that there is a spontaneous production of entropy on the solar turbulent convection. From the study of the statistics of the vertical heat flux we have found a strong indication that the solar turbulent convection in non-magnetic regions satisfies the symmetries predicted by the FR of the Gallavotti–Cohen fluctuation theorem, similarly to the systems studied in [10,13].

In conclusion, here, we have provided a first indication for the validity of the prediction of the Gallavotti and Cohen fluctuation theorem in the framework of a real astrophysical system.

## Abbreviations

The following abbreviations are used in this manuscript:

NESS	Non Equlibrium Stationary States
FR	Fluctuation Relation
GCFR	Gallavotti–Cohen Fluctuation Relation
LoS	Line-of-Sight
NSO	National Solar Observatory
DST	Dunn Solar Telescope
IBIS	Interferometric BIdimensional Spectropolarimeter
FoV	Field-of-View
CoG	Center of Gravity
PDFs	Probability Density Functions
